# The risk of late or advanced presentation of HIV infected patients is still high, associated factors evolve but impact on overall mortality is vanishing over calendar years: results from the Italian MASTER Cohort

**DOI:** 10.1186/s12889-016-3477-z

**Published:** 2016-08-25

**Authors:** Elena Raffetti, Maria Concetta Postorino, Francesco Castelli, Salvatore Casari, Filippo Castelnuovo, Franco Maggiolo, Elisa Di Filippo, Alessandro D’Avino, Andrea Gori, Nicoletta Ladisa, Massimo Di Pietro, Laura Sighinolfi, Fabio Zacchi, Carlo Torti

**Affiliations:** 1 Institute of Hygiene, Epidemiology and Public Health, University of Brescia, Brescia, Italy; 2 Department of Medical and Surgical Sciences, Unit of Infectious and Tropical Diseases, University “Magna Graecia”, Catanzaro, Italy; 3 Institute of Infectious and Tropical Diseases, University of Brescia, Brescia, Italy; 4 Infectious Diseases, Spedali Civili Hospital, Brescia, Italy; 5 Clinic of Infectious Diseases, “Papa Giovanni XXIII” Hospital, Bergamo, Italy; 6 Institute of Infectious Diseases, Catholic University of Sacred Heart, Rome, Italy; 7 Clinic of Infectious Diseases, Ospedale “S. Gerardo”, Monza, Italy; 8 Institute of Infectious Diseases, University of Bari, Bari, Italy; 9 Clinic of Infectious Diseases, “S. M. Annunziata” Hospital, Florence, Italy; 10 Clinic of Infectious Diseases, “S. Anna” Hospital, Ferrara, Italy; 11 Clinic of Infectious Diseases, Istituti Ospitalieri Cremona, Cremona, Italy

**Keywords:** HIV infection, AIDS, Late diagnosis, Late presentation, Advanced HIV disease

## Abstract

**Background:**

We aimed at evaluating frequency and factors associated with late presentation and advanced HIV disease and excess risk of death due to these conditions from 1985 to 2013 among naïve HIV infected patients enrolled in the Italian MASTER Cohort.

**Methods:**

All antiretroviral naive adults with available CD4+ T cell count after diagnosis of HIV infection were included. Multivariable logistic regression analysis investigated factors associated either with late presentation or advanced HIV disease. Probabilities of survival were estimated both at year-1 and at year-5 according to the Kaplan-Meier method. Flexible parametric models were used to evaluate changes in risk of death overtime according to late presentation and advanced HIV disease. The analyses were stratified for calendar periods.

**Results:**

19,391 patients were included (54 % were late presenters and 37.6 % were advanced presenters). At multivariable analysis, the following factors were positively associated with late presentation: male gender (OR = 1.29), older age (≥55 years *vs*. <25 years; OR = 7.45), migration (OR = 1.54), and heterosexual risk factor for HIV acquisition (OR = 1.52) or IDU (OR = 1.27) compared to homosexual risk. Survival rates at year-5 increased steadily and reached 92.1 % for late presenters *vs*. 97.4 % for non-late presenters enrolled in the period 2004–2009. Using flexible parametric models we found a sustained reduction of hazard ratios over time for any cause deaths between late and non-late presenters over time. Similar results were found for advanced HIV disease.

**Conclusion:**

Screening polices need to be urgently implemented, particularly in most-at-risk categories for late presentation, such as migrants, older patients and those with heterosexual intercourse or IDU as risk factors for HIV acquisition. Although in recent years the impact of late presentation on survival decreased, about 10 % of patients diagnosed in more recent years remains at increased risk of death over a long-term follow-up.

**Electronic supplementary material:**

The online version of this article (doi:10.1186/s12889-016-3477-z) contains supplementary material, which is available to authorized users.

## Background

In 2011 consensus definitions of late presentation and advanced HIV disease were formulated [1]. Late presenters were defined as patients diagnosed with a CD4+ T cell count <350/mm^3^ or with an AIDS defining event. About 50 % of HIV infected patients in Europe were late presenters [1, 2]. Different HIV transmission patterns were reported in late *vs*. non-late presenters, although late presentation remains a major problem in any HIV exposure groups [1, 3]. Indeed, rates of late presentation in 2010–2013 were higher in Central Europe (49.8 %), followed by Northern (48.8 %), Southern (45.8 %) and Eastern Regions (38.3 %), especially among intravenous drug users (IDUs) [3, 4].

Late presentation is associated with a greater risk of polypharmacy, drug non adherence and suboptimal virological and immunological effectiveness of antiretroviral therapy [5–7]. From a pharmaco-economical point of view, late presentation increases costs for health care [5]. Patients presenting late show higher rates of AIDS and death when compared with subjects presenting earlier [6, 8, 9], especially during the first year after HIV diagnosis [1]. Moreover, from a public health perspective, people presenting late were spending longer time being unaware of their status, did not start any combination antiretroviral therapy (cART), which is effective in reducing HIV RNA (thereby reducing the risk of transmission by >95 % [10]), so late presentation contributes to enhance rates of HIV transmission [11]. Indeed, some studies reported that community HIV RNA concentrations were correlated with incedence of new HIV diagnoses in specific contexts (e.g., among IDUs or men who have sex with men, MSM) [12, 13]. For these reasons, the World Health Organization (WHO), promotes initiatives to increase HIV testing and retention into care, aimed at reducing rates of late diagnosis and consequently HIV transmission [14], but in reality such campaigns are poorly implemented in some contexts.

In Italy, people presenting late were older (age >50 years), migrants, and more frequently were diagnosed in the Southern-Central Regions [15, 16]. Italian late presenters acquired HIV infection most frequently by heterosexual contacts [15, 16], and secondly by IDU, whereas in other European Countries higher rates of late presenters were reported among IDUs [17].

Until now, national studies evaluated late presentation in a limited period [15, 18, 19]. Moreover, regional analyses were performed along a extended time of observation, but sample was not representative of the national scenario of late presentation and size of the population did not allow to perform a stratified analysis by calendar periods [20, 21]. So, we believe that the available results are not powerful enough to drive preventative strategies and monitor the effects of late presentation on patient survival.

The aim of this study was to study late presentation in a large national Italian cohort from the beginning of the epidemic in Italy to present days. In particular, we evaluated frequency of late presentation over calendar years and associated factors. We further examined whether late presentation was associated with patient survival, and if differences in the mortality rates between late presenters and non late presenters improved overtime to have indications on possible gaps in HIV disease management that may be still present in these patients.

## Methods

HIV positive patients enrolled in the Italian MASTER (Standardized Management of Antiviral Therapy) Cohort [22] from January 1985 to December 2013 were selected. Epidemiological, virological, immunological, demographical and clinical data from all patients in the Cohort are stored and annually updated into a common database (Health & Notes 3.5^®^, Healthware S.p.A., Naples, Italy).

All antiretroviral treatment-naïve patients with available CD4+ T cell count at enrolment were included in the study. CD4+ T cell count at enrolment was defined as CD4+ T cell count closest to and within 6 months since enrolment. Viral load was available since 1996 and defined as HIV RNA closest to or within 30 days since enrolment, including only treatment-naïve subjects at enrolment.

Late presentation refers to people diagnosed with HIV with a CD4+ T cell count below 350/mm^3^ or with an AIDS defining event regardless of the CD4+ T cell count in the 6 months following HIV diagnosis. Advanced HIV disease refers to people diagnosed with HIV with a CD4+ T cell count below 200/mm^3^ or with an AIDS defining event, regardless of CD4+ T cell count in the 6 months following HIV diagnosis. HCV co-infection was defined on the basis of positive HCV antibodies.

Vital status and date of death were ascertained through clinical charts, and through a record-linkage with Local Health Authority mortality registers in about one third of patients.

### Statistical analysis

Follow-up was determined from enrolment to 31 December 2013 or last follow-up visit, or death, whichever occurred first.

Differences in demographic and clinical features of patients were tested using common statistical methods for comparisons of means and proportions. Associations of demographical and clinical features with late presentation and advanced HIV disease were investigated using a logistic regression model, providing estimates of the odds ratios (ORs) as measures of association and their 95 % confidence intervals (95 % CIs). We adjusted the models for the following a priori confounders: gender, age at enrolment, country of origin, HIV exposure category and period of enrolment as covariates. Separate analyses have been performed in patients presenting at different time periods. The probabilities of survival were estimated at year-1 and at year-5 according to the Kaplan-Meier methods according to late presentation, advanced HIV disease and time periods. The survival curves were compared using the log-rank test. As sensitivity analysis, we calculated probabilities of survival at year-1 and at year-5 applying an inverse probability weighted method to the Kaplan-Meier curves in order to adjust for selection bias due to lost to follow-up (365 days and over since their last visit or exams). Cubic splines were modeled using flexible parametric models [23–25] to evaluate changes of risk of death overtime between late presenters and non late presenters and between subjects with and without advanced HIV disease. The models were adjusted for a priori confounders, including age, gender, country of origin and HIV exposure category. The results were expressed in terms of hazard ratios (HRs) over time and their 95%CIs.

All statistical tests were two-sided, assuming a level of significance of 0.05 and were performed using Stata software version 12.0 (Stata Corp, College Station, TX, USA).

## Results

### Overall population

19,391 patients were included in the study contributing 143,097 person-years (median 5.2 years of follow-up). Among these patients, 10,486 (54.1 %) were late presenters and 7,291 (37.6 %) had advanced HIV disease at presentation. Table 1 reports the main demographic, epidemiological and clinical characteristics.

**Table 1:** Characteristics of subjects at enrolment according to late presentation

Subjects’ characteristics	No	Yes	Total	*P* value
	*n* (%)*	*n* (%)^a^	*n* (%)^b^	
Total	8905	10486	19391	
Gender				
Male	6477 (44.7)	8022 (55.3)	14499 (74.8)	<0.001
Female	2428 (49.6)	2464 (50.4)	4892 (25.2)	
Age at enrolment (years)				
< 25	1869 (71.9)	729 (28.1)	2598 (13.4)	<0.001
25–34	4362 (49.3)	4478 (50.7)	8840 (45.6)	
35–44	1863 (36.2)	3290 (63.9)	5153 (26.6)	
45–54	602 (30.8)	1355 (69.2)	1957 (10.1)	
≥ 55	209 (24.8)	634 (75.2)	843 (4.3)	
Mean age, years (SD)	32.1 (8.9)	37.0 (9.9)	34.8 (9.8)	<0.001
Mean CD4 T+ cell count//mm^3^ (SD)	644.4 (275)	171.1 (137.4)	403.1 (320.4)	<0.001
Period of enrolment				
1985–1991	2612 (60.8)	1681 (39.2)	4293 (22.1)	<0.001
1992–1997	2010 (41.3)	2855 (58.7)	4865 (25.1)	
1998–2003	1727 (40.6)	2528 (59.4)	4255 (21.9)	
2004–2009	1642 (42.5)	2217 (57.5)	3859 (19.9)	
2010–2013	914 (43.1)	1205 (56.9)	2119 (10.9)	
Country of origin				
Available^b^	8738 (98.1)	10210 (97.4)	18948 (97.7)	<0.001
Italy	7996 (47.1)	8991 (52.9)	16987 (89.4)	
Others	742 (37.8)	1219 (62.2)	1961 (10.3)	
HIV exposure category				
Available^b^	8156 (91.6)	9528 (90.9)	17684 (91.2)	<0.001
MSM	1333 (47.8)	1455 (52.2)	2788 (15.8)	
IDUs	3879 (51.5)	3654 (48.5)	7533 (42.6)	
Heterosexuals	2337 (39.2)	3620 (60.8)	5957 (33.7)	
MSM-IDUs	60 (57.1)	45 (42.9)	105 (0.6)	
Heterosexuals-IDUs	256 (41.0)	368 (59.0)	624 (3.5)	
Haemophilia/Perinatal transmission	26 (53.1)	23 (46.9)	49 (0.3)	
Unknown	32 (48.5)	34 (51.5)	66 (0.4)	
Others	233 (41.5)	329 (58.5)	562 (3.2)	
Viral load at enrolment^c^				
Available^b^	3678 (41.3)	5348 (51.0)	9026 (46.5)	<0.001
Mean HIV RNA, log_10_ copies/ml (SD)	4.2 (1.0)	4.9 (0.9)	4.6 (1.0)	
Hepatitis C co-infection				
Available^b^	6913 (77.6)	6923 (66.0)	13836 (71.4)	<0.001
No	3250 (45.2)	3942 (54.8)	7192 (52.0)	
Yes	3663 (55.1)	2981 (44.9)	6644 (48.0)	
Hepatitis B co-infection				
Available^b^	7832 (88.0)	7836 (74.7)	15668 (80.8)	<0.001
No	6889 (50.8)	6669 (49.2)	13558 (86.5)	
Yes	943(44.7)	1167 (55.3)	2110 (13.5)	

^a^Row percentages ^b^column percentages. ^c^Viral load is available since 1996 and defined as viral load closest to and within 30 days since enrolment, including only treatment-naïve subjects at enrolment. CD4+ T cell count at enrolment is defined as CD4+ T cell count closest to and within 6 months since enrolment. *IDUs* Intravenous drug users, *MSM* Men who have sex with men

Percentages of late presenters increased from 1985–1991 (39.2 %) to 1992–1997 (58.7 %) and then remained stable over time (56.9 % in 2010–2013). Likewise, the prevalence of advanced HIV disease at presentation increased from 1985–1991 (26.9 %) to 1992–1997 (42.1 %), and then showed a modest reduction up to 2010–2013 (38.7 %). The cumulative probability of loss to follow-up at year-3 was 21.4 % (95 % CI 20.8-22.0 %).

Most patients were Italians (89.4 %) and males (74.8 %). Frequency of males ranged from 77.7 to 71.8 % among observational periods. Mean age was 34.8 years (standard deviation, SD 9.8). Major risk factors for HIV acquisition were IDU (42.6 %) and sexual transmission (15.8 % MSM, 33.7 % heterosexuals). In particular, for males the major risk factors were in the order: IDU (46.7 %), heterosexual transmission (24.8 %) and MSM (21 %). For females the major risk factors were: heterosexual transmission (59.7 %) and IDU (30.5 %). Fourty-eight percent patients had HCV co-infection; while 13.5 % patients were co-infected by HBV. Prevalence of HCV co-infection decreased steadily, from 79.2 % in 1985–1991 to 20.3 % in 2010–2012, whereas prevalence of HBV remained stable between 10 % and 19.1 % throughout the entire study period.

Considering cART, no prescription, two nucleoside reverse-transcriptase inhibitors plus one non-nucleoside reverse-transcriptase inhibitors (2NRTIs + NNRTI) and 2NRTIs *plus* one protease inhibitor either boosted or unboosted by ritonavir (2NRTIs + PI ± r) in the periods under study were, respectively: a) 1985–1991: 51, 0 and 0 %, b) 1992–1997: 45.6, 3.2 and 15.5 %; c) 1998–2003: 5.2, 29.9 and 41.6 %; d) 2004–2009: 1.6, 31.2 and 45.7 %; e) 2010–2013: 0.4, 16.5 and 37.7 %.

Table 1 describes demographic and clinical features according to late presentation. Subjects with foreign origins had more frequently late presentation than Italians (62.2 *vs*. 52.9 %, *p*<0.001). Patients presenting late were older than non-late presenters [mean age of late presenters was 37 years (SD 9.9) *vs*. 32 years (SD 8.9) for non late presenters]. Mean CD4+ T cell count was 171/mm^3^ (SD 137.4) and 644.4/mm^3^ (SD 275) in late and non-late presenters, respectively. Subjects with heterosexual risk factor had a late presentation more frequently than MSM and IDUs (60.8 *vs*. 52.2 % and 48.5 %, *p* < 0.001). Prevalence of late presenters among MSM decreased from 58.7 % in 1985–1991 to 44.9 % in 2010–2013, whereas, among subjects with heterosexual intercourse as risk factor for HIV acquisition, it increased from 37.2 % in 1985–1991 to 58.2 % in 1992–1997 and to 65.7 % in 2010–2013. HCV co-infected patients were less frequently late presenters than non-HCV co-infected (44.9 *vs.* 54.8 %, *p*<0.001), whereas HBV co-infected had an higher prevalence of late presentation than individuals not co-infected by HBV (55.3 *vs*. 49.2 %, *p*<0.001).

### Factors associated with late presentation

As shown in Table 2, when compared with MSM, the following categories were more likely to present late: people of male gender (OR = 1.29), with older age (≥55 years *vs.* <25 years; OR = 7.45), migrants (OR = 1.54), heterosexuals (OR = 1.52), and IDUs (OR = 1.27).

**Table 2:** Multivariable logistic regression model: association of demographical and clinical features with late presentation according to observation period

		Total period (*n* = 17333)		1985–1991 (*n* = 4254)		1992–1997 (*n* = 4614)		1998–2003 (*n* = 3892)		2004–2009 (*n* = 3142)		2010–2013 (*n* = 1431)	
Variable	Category	ORs (95 % CI)	*P* value	ORs (95 % CI)	*P* value	ORs (95 % CI)	*P* value	ORs (95 % CI)	*P* value	ORs (95 % CI)	*P* value	ORs (95 % CI)	*P* value
Gender	Male vs Female	1.29 (1.19–1.40)	<0.001	1.18 (1.00–1.40)	0.054	1.45 (1.25–1.69)	<0.001	1.31 (1.12–1.54)	0.001	1.17 (0.97–1.42)	0.099	1.10 (0.82–1.46)	0.525
Age at enrollment	<25	Ref.		Ref.		Ref.		Ref.		Ref.		Ref.	
	25–34	2.44 (2.21–2.70)	<0.001	2.55 (2.20–2.97)	<0.001	2.99 (2.42–3.69)	<0.001	2.52 (1.88–3.39)	<0.001	1.48 (1.10–2.00)	0.009	1.58 (1.04–2.40)	0.032
	35–44	4.11 (3.65–4.61)	<0.001	5.33 (4.04–7.02)	<0.001	4.84 (3.82–6.14)	<0.001	4.44 (3.29–6.01)	<0.001	2.49 (1.84–3.35)	<0.001	2.65 (1.74–4.03)	<0.001
	45–54	5.79 (4.98–6.74)	<0.001	5.04 (2.90–8.73)	<0.001	5.20 (3.68–7.35)	<0.001	7.70 (5.35–11.08)	<0.001	3.57 (2.56–4.99)	<0.001	3.75 (2.39–5.90)	<0.001
	≥55	7.45 (6.06–9.16)	<0.001	21.65 (6.07–77.22)	<0.001	8.06 (5.19–12.53)	<0.001	10.08 (6.27–16.19)	<0.001	4.19 (2.76–6.36)	<0.001	3.98 (2.29–6.94)	<0.001
Country of Origin	Others vs Italy	1.54 (1.38–1.73)	<0.001	1.91 (1.04–3.49)	0.036	0.94 (0.67–1.33)	0.742	1.53 (1.25–1.88)	<0.001	1.48 (1.21–1.81)	<0.001	1.52 (1.15–2.03)	0.004
HIV exposure category	MSM	Ref.		Ref.		Ref.		Ref		Ref.		Ref.	
	IDUs	1.27 (1.15–1.40)	<0.001	0.59 (0.43–0.80)	0.001	1.44 (1.18–1.76)	<0.001	1.27 (1.04–1.56)	0.017	1.18 (0.93–1.50)	0.172	2.12 (1.30–3.47)	0.003
	Heterosexuals	1.52 (1.37–1.69)	<0.001	0.54 (0.37–0.77)	0.001	1.44 (1.15–1.81)	0.002	1.69 (1.39–2.06)	<0.001	1.73 (1.43–2.10)	<0.001	1.89 (1.42–2.50)	<0.001
	MSM-IDUs	0.89 (0.59–1.34)	0.584	0.44 (0.21–0.93)	0.032	0.69 (0.34–1.38)	0.294	0.86 (0.32–2.30)	0.77	2.47 (0.61–9.97)	0.204	2.89 (0.25–32.84)	0.393
	Heterosexuals-IDUs	1.88 (1.56–2.26)	<0.001	1.47 (0.95–2.27)	0.085	2.39 (1.73–3.29)	<0.001	1.42 (0.93–2.17)	0.106	0.67 (0.40–1.11)	0.121	1.07 (0.4–2.88)	0.896
	Haemophilia /Perinatal transmission	0.84 (0.36–1.09)	0.574	0.26 (0.09–0.76)	0.014	0.55 (0.17–1.72)	0.302	1.73 (0.32–9.40)	0.528	1.43 (0.08–24.65)	0.808	2.01 (0.43–9.38)	0.373
	Unknown	1.05 (0.42–1.10)	0.856	0.95 (0.14–6.53)	0.958	1.21 (0.43–3.36)	0.718	1.89 (0.16–22.73)	0.617	1.50 (0.52–4.36)	0.456	0.79 (0.35–1.81)	0.582
	Others	1.43 (0.83–1.20)	0.001	0.97 (0.49–1.90)	0.92	1.09 (0.71–1.66)	0.702	1.21 (0.79–1.85)	0.372	2.37 (1.53–3.67)	<0.001	1.58 (0.99–2.53)	0.055
Period of enrollment	1985–1991	Ref.											
	1992–1997	1.56 (1.43–1.71)	<0.001										
	1998–2003	1.29 (1.16–1.43)	<0.001										
	2004–2009	1.14 (1.02–1.28)	0.026										
	2010–2013	1.17 (1.01–1.35)	0.032										

The associations of demographical and clinical features with late presentation were investigated using a logistic regression model, providing estimates of the odds ratios (ORs) as measures of association. Separate analyses have been performed in different time periods. 95 % *CI* 95 % confidence interval, *OR* odds ratio. *Ref* reference category

In the time-stratified analysis, statistically significant associations between late presentation and male gender were found in all periods, but not in the more recent years (2004–2009 and 2010–2013). Age >25 years and migration were associated with late presentation in any time periods. Heterosexual transmission and IDU compared to MSM were associated with higher risk of late presentation in almost all time periods.

We also found associations when we evaluated factors related with advanced HIV disease (see Additional file 1: Table S1). In the overall population, advanced HIV disease was positively associated with male gender [OR = 1.47, 95%CI 1.35–1.60], older age at enrolment (≥55 years *vs*. <25 years; OR = 9.59, 95%CI 7.83–11.75), migration (OR = 1.39, 95%CI 1.24–1.56) and heterosexual intercourse (OR = 1.44, 95%CI 1.30–1.60) or IDU (OR = 1.17, 95%CI 1.05–1.29) as risk factor for HIV acquisition compared to MSM.

### Survival analysis stratified along calendar years

**Fig. 1:**
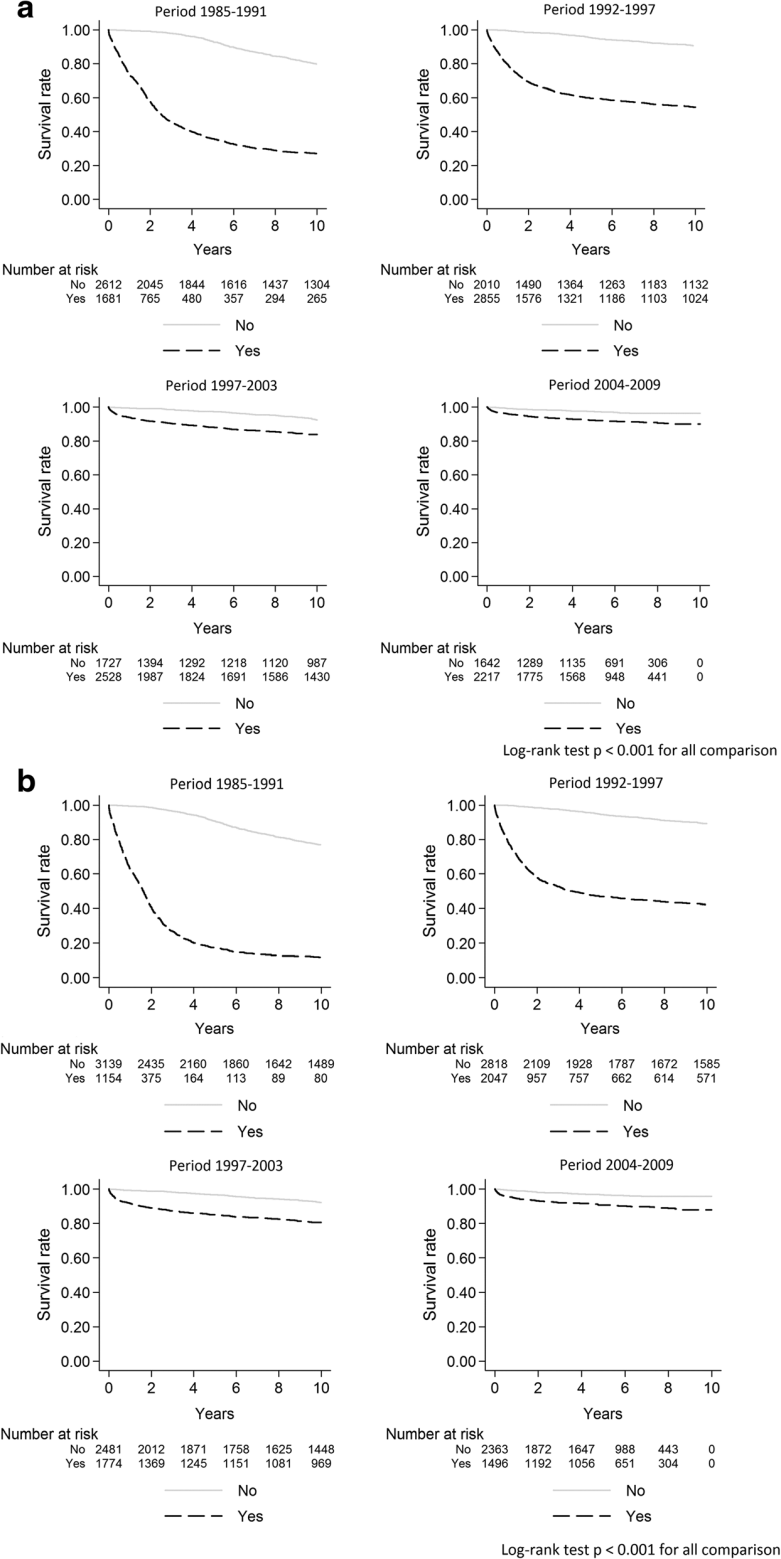
Survival rates from 1985 to 2009 according to late presentation (**a**) and advanced HIV disease (**b**)

**Fig. 2:**
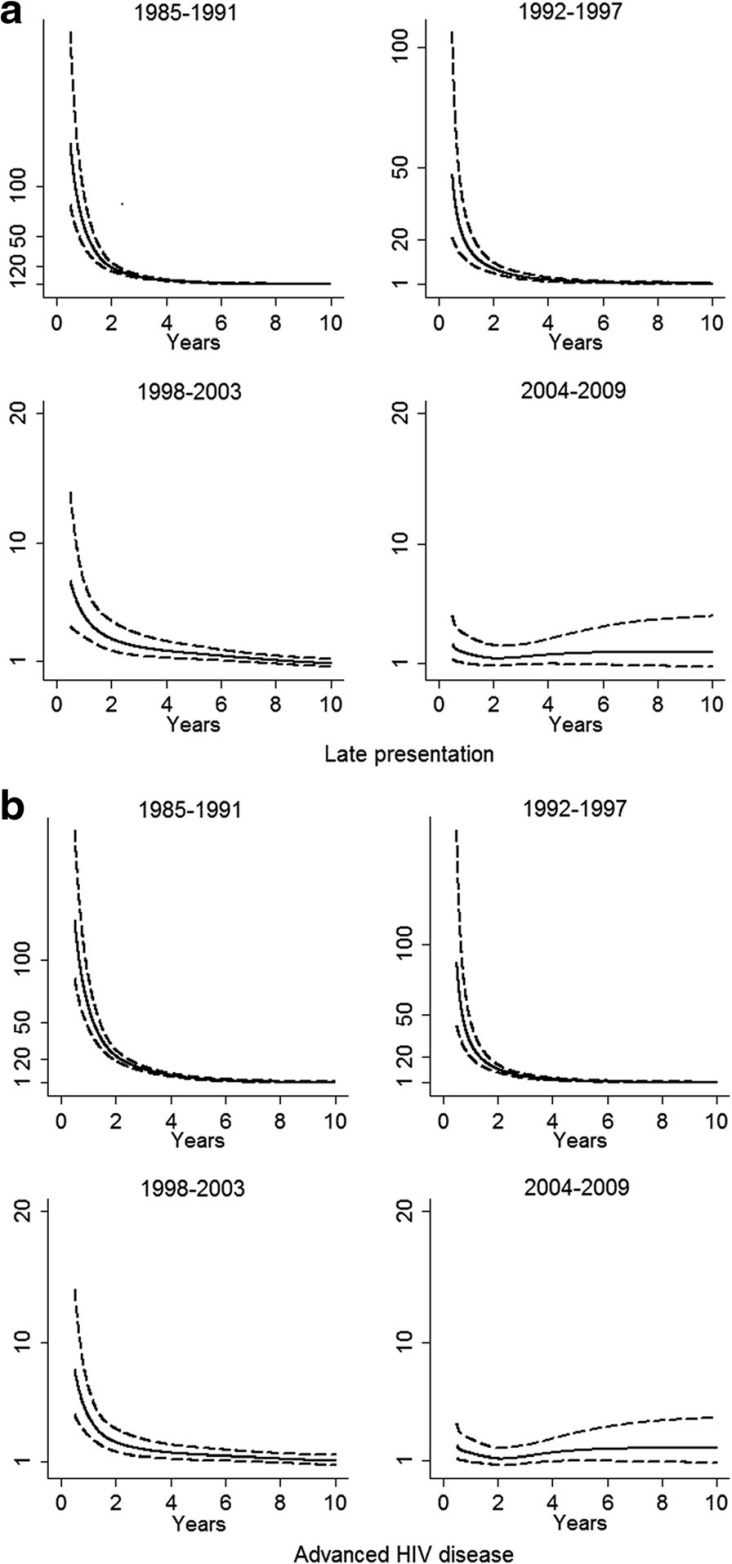
Hazard ratios over time for late presentation (**a**) and advanced HIV disease (**b**) using flexible parameter models

Survival estimates for people presenting late compared to non-late presenters were 73.9 *vs.* 99.6 % at year-1 and 35.8 *vs*. 93.3 % at 5-year in 1985–1991 (log-rank test *p*<0.001 for both comparisons). Survival rates increased steadily in 1992–1997 and in 1998–2003 either for late presenters or for non late presenters. Survival rates at year-1 and at year-5 reached 95.9 and 92.1 % for late presenters *vs.*99.2 and 97.4 % for non-late presenters in 2004 to 2009 but differences between late and non late presenters remained significant for both comparisons (*p *<0.001). (Fig. 1a). Similarly, a better survival was observed in subjects presenting without advanced HIV disease than in subjects with advanced HIV disease (log-rank test *p*< 0.001 for the whole period) (Fig. 1b). Survival rates at year-1 and at year-5 increased in subjects without advanced HIV disease at presentation from 99.5 and 90.8 % in 1985–1991 to 99 and 96.6 % in 2004–2009. For subjects with advanced HIV disease at presentation, survival rates at year-1 and at year-5 increased from 62.8 and 17.2 % in 1985–1991 to 94.6 and 90.8 % in 2004–2009. When we calculated the survival estimates at year-1 and at year-5 using a model weighted for losses to follow-up, the survival estimates for late-presenters and subjects with advanced HIV disease were lower in 1985–1991 and in 1992–1997 than non-weighted survival estimates (see Additional file 1: Table S2). Late presentation and advanced HIV disease were also evaluated in multivariate flexible parametric models with restricted cubic-splines for the HRs of these variables, which showed a sustained reduction of HRs over time in all periods (Fig. 2). Moreover, when mortality at year-1 was studied in the same models, HRs for late presenters* vs*. non late presenters were 70.4 (95%CI 46–108) in 1985–1991, 22.1 (95%CI 13.8–35.4) in 1992–1997, 5.4 (95%CI 3.5–8.3) in 1998–2003 and 2.5 (95%CI 1.5–4.3) in 2004–2009.

## Discussion

In the present study we evaluated factors associated with late presentation and compared risk of death for late presenters with risk of death for non late presenters from 1985 to 2013 in a large cohort of drug naïve HIV infected subjects enrolled in Italy. These analyses were also stratified for calendar periods.

Overall, proportion of late presentation and advanced HIV disease were 54 and 38 %, respectively. Late diagnoses were more frequent in the period from 1992 to 2009. Proportion of late presenters in our cohort is in line with previous studies reported by Collaboration of Observational HIV Epidemiological Research Europe Study (COHERE) for Southern Europe in the same time frame [1, 3] and by other recent European and Italian studies [15–21, 26–29]. This was not unexpected because the Italian MASTER Cohort is one of the main contributors to COHERE. Also, profile of people presenting late was similar to previous European and Italian estimates [1, 3, 15]. Indeed, patients presenting late were mostly males, migrants and older than individuals who presented earlier. Only in specific contexts female patients accounted for the majority of late presentations, especially because of the female component of the migrant population coming from Sub Saharian Africa [30–32].

We assessed evolution of factors associated with late presentation along calendar periods. In parallel with a decrease of late presentation among MSM over calendar periods, there was a increase in prevalence of late presentation among heterosexuals, and heterosexual transmission was one of the main risk factors for late presentation from 1992 to 2013. So, we could hypothesize that information and screening polices were quite successful among MSM communities, although a increase of HIV diagnoses in young MSM has been recently reported [33]. The most likely explanation is that older MSM had personal experiences of HIV complications occurring in friends, partners or members of the same community, while younger MSM may only be informed through preventative campaigns in the field. Although the youngest MSM may have not been involved or not respondent to these campaigns as demonstrated by an increase of new HIV diagnoses in the same population in the latest years [33], the level of information in MSM may be higher than in other populations. Moreover, information campaigns in MSM were proven to be successful [34], while heterosexuals did not regularly practice safe sex even if they were well informed about prevention of HIV or other sexually transmitted diseases [35]. It is difficult to explain this possible discrepancy but general differences in the cultural level or background of experiences may play a role.

Compared to MSM, IDUs had a greater risk of late presentation, consistently in all calendar periods. This result has to be referred to syringe exchange (especially in Italy where programs to provide syringes to IDUs are lacking). However, even in IDU populations the risk of HIV acquisition appeared to be more linked with heterosexual intercourses than with IDU by itself [36]. Also, use of recreational drugs (even not intravenous) may be responsible for a increased risk of transmission through sexual intercourses independently from gender and types of sexual relationships. Therefore, further behavioral studies should be conducted to understand the actual ways of HIV transmission and behaviors that may enhance this risk.

Overall, the risk of late presentation was frequently associated with heterosexual transmission in more recent years. Lack of knowledge and underestimation of HIV transmission through this route probably contributed to a late diagnosis, especially among heterosexuals. Indeed, the main reason for late HIV testing reported by heterosexual late presenters was unawareness of risk or unawareness of symptoms of HIV [32]. People presenting late often declare that they were not ill, although the majority of them were symptomatic during the year preceding HIV diagnosis and consulted a physician for these symptoms [32]. In other cases (as among foreign patients, women and IDUs), socioeconomic status and poor access to health care may have contributed to late presentation [37, 38]. At the same time, it is possible that some other factors may contribute to decreased use of condoms, either among heterosexuals or MSM, as alcohol abuse and/or illicit drug use. So, informative campaigns for prevention and early diagnoses should be targeted to persons with any risk behaviors (including heterosexual individuals) rather than to the classical risk categories, such as IDUs or MSM.

In our study we found that survival rates at year-1 and at year-5 increased steadily either for late presenters or for non late presenters. However, survival was always poorer in late presenters compared to non late presenters in all time periods. Using flexible parametric models we found that the excess mortality rate in late presenters was highest immediately after diagnosis and then declined, mostly in farthest years (from 1985 to 1998). Similar results were found for people with advanced HIV disease. In more recent years, improved standards of care and more effective antiretroviral therapies were introduced [39], so this improved clinical outcomes even in patients who presented late. This consideration is important because if the definition of late presentation is applied to classify patients by clinical risk, the existing definition may be anachronistic and should be somehow revised. Alternatively, the improved clinical outcome in late presenters may be due to different composition of the patient cohort across calendar periods. If this is true, the definition should be made more specific in different categories of patients. Notwithstanding these considerations, late presentation should be avoided anyway because it has a strong negative impact on test-and-treat strategies promoting HIV transmission. Also, anticipation of therapy at higher CD4 T cell counts improves patient survival as demonstrated by the START study [40], so more recent guidelines recommend to start cART irrespectively of CD4 T cell count at diagnosis [41]. Moreover, earlier cART reduces morbidities [40], which was not an outcome of our study.

Several limitations of our work should be considered. First, we likely underestimated late presenters in farthest periods, because people diagnosed at that time may have died after the access to the hospital and not survived long enough to be enrolled in the cohort. Second, transient low CD4 T+ cell count in patients with recent infections may lead to overestimation of late presenters, classified according to the consensus definition [42]. However, percentage of patients with recent infection in our cohort was very small and did not influence the results. Indeed, people with a diagnosis of acute or recent infection were very few in the cohort (<1 % in any calendar periods under study). Furthermore, our cohort was affected by a high cumulative probability of loss to follow-up at year-3. This high rate of loss to follow-up is common in retrospective cohort studies, however. Selection bias due to loss to follow up represents a threat to the internal validity of the survival estimates and unadjusted model could overestimated the survival estimates. In our paper we tried to mitigate this selection bias presenting also inverse probability-of-censoring weighted survival estimates.

## Conclusions

In conclusion, late presentation affected over 50 % of HIV diagnoses in our cohort. Combination antiretroviral therapy and improved health care contributed to reduce short and long term mortality among late presenters in more recent years. Interestingly, we found that the excess risk of death due to late presentation on overall mortality (at year-1 and at year-5) decreased over calendar years. However, statistically significant differences in survival rates between late and non late presenters were found in all time periods. IDUs, people with heterosexual intercourses, migrants and older patients were more at risk of presenting late in our cohort. Similar associations were found for advanced HIV disease. However, results obtained in the overall populations have to be carefully interpreted, because factors associated with late presentation may change over time, as showed by our analysis stratified by calendar years. Further strategies to encourage and facilitate earlier diagnosis are needed: widespread testing, reaching vulnerable populations, identify and fight against stigma. Increasing HIV testing, information campaigns about HIV risk factors and improvement of the test-and-treat strategy recommended by in the Italian Guidelines only recently [41] will hopefully reduce rates of HIV transmission and late presentations. So our results should be interpreted as a baseline analysis for further monitoring of patient characteristics at diagnosis and clincal outcomes over the long term.

## Additional file

Additional file 1: Table S1. Multivariable logistic regression model: association of demographical and clinical features with advanced HIV disease according to observation period. **Table S2.** Survival rate at year-1 and at year-5 from 1985 to 2009 according to late presentation and advanced HIV disease (weighted for losses to follow-up). (DOCX 23 kb)

## Abbreviations

AIDS: Acquired Immune Deficiency Syndrome
cART: Combination Antiretroviral Therapy
HBV: Hepatitis B Virus
HCV: Hepatitis C Virus
HIV: Human Immuno-Deficiency Virus
IDU: Intravenous Drug Use
IDUs: Intravenous Drug Users
MASTER Cohort: Standardized Management of Antiviral Therapy Cohort
MSM: Men Have Sex With Men
