# Supplementary material for: The risk of late or advanced presentation of HIV infected patients is still high, associated factors evolve but impact on overall mortality is vanishing over calendar years: results from the Italian MASTER Cohort
Source: BMC Public Health. 2016 Aug 25;16(1):878. doi: 10.1186/s12889-016-3477-z (PMC4997689; doi:10.1186/s12889-016-3477-z)
Supplement: Additional file 1: Table S1. — Multivariable logistic regression model: association of demographical and clinical features with advanced HIV disease according to observation period. Table S2. Survival rate at year-1 and at year-5 from 1985 to 2009 according to late presentation and advanced HIV disease (weighted for losses to follow-up). (DOCX 23 kb) [file 12889_2016_3477_MOESM1_ESM.docx]

|  |  | **Total period (n=17333)** | | **1985-1991 (n=4254)** | | **1992-1997 (n=4614)** | | **1998-2003 (n=3892)** | | **2004-2009 (n=3142)** | | **2010-2013 (n=1431)** | |
| --- | --- | --- | --- | --- | --- | --- | --- | --- | --- | --- | --- | --- | --- |
| **Variable** | **Category** | **ORs (95%CI)** | **P value** | **ORs (95%CI)** | **P value** | **ORs (95%CI)** | **P value** | **ORs (95%CI)** | **P value** | **ORs (95%CI)** | **P value** | **ORs (95%CI)** | **P value** |
| **Gender** | Male *vs.* Female | 1.47 (1.35-1.60) | <0.001 | 1.38 (1.13-1.68) | 0.002 | 1.55 (1.33-1.82) | <0.001 | 1.49 (1.27-1.76) | <0.001 | 1.48 (1.22-1.80) | <0.001 | 1.32 (1.00-1.75) | 0.049 |
| **Age at enrollment** | <25 | Ref. |  | Ref. |  | Ref. |  | Ref. |  | Ref. |  | Ref. |  |
|  | 25-34 | 2.63 (2.33-2.98) | <0.001 | 2.69 (2.25-3.22) | <0.001 | 3.04 (2.36-3.92) | <0.001 | 2.81 (1.95-4.06) | <0.001 | 1.76 (1.22-2.52) | 0.002 | 1.71 (1.06-2.78) | 0.029 |
|  | 35-44 | 4.61 (4.03-5.27) | <0.001 | 5.35 (4.02-7.12) | <0.001 | 5.12 (3.90-6.72) | <0.001 | 4.66 (3.22-6.76) | <0.001 | 3.33 (2.32-4.76) | <0.001 | 2.93 (1.82-4.72) | <0.001 |
|  | 45-54 | 6.32 (5.39-7.42) | <0.001 | 7.38 (4.23-12.87) | <0.001 | 6.61 (4.61-9.49) | <0.001 | 7.62 (5.06-11.46) | <0.001 | 4.18 (2.85-6.13) | <0.001 | 3.57 (2.17-5.87) | <0.001 |
|  | ≥55 | 9.59 (7.83-11.75) | <0.001 | 19.26 (6.60-56.17) | <0.001 | 11.47 (7.40-17.78) | <0.001 | 10.71 (6.63-17.29) | <0.001 | 7.15 (4.58-11.17) | <0.001 | 4.32 (2.42-7.70) | <0.001 |
| **Country of Origin** | Others *vs*. Italy | 1.39 (1.24-1.56) | <0.001 | 1.14 (0.58-2.24) | 0.700 | 0.80 (0.56-1.15) | 0.228 | 1.36 (1.11-1.66) | 0.003 | 1.45 (1.18-1.78) | <0.001 | 1.57 (1.19-2.07) | 0.001 |
| **HIV exposure category** | MSM | Ref. |  | Ref. |  | Ref. |  | Ref. |  | Ref. |  | Ref. |  |
|  | IDUs | 1.17 (1.05-1.29) | 0.004 | 0.47 (0.35-0.63) | <0.001 | 1.26 (1.03-1.54) | 0.025 | 1.21 (0.99-1.48) | 0.066 | 1.40 (1.09-1.80) | 0.008 | 2.40 (1.50-3.85) | <0.001 |
|  | Heterosexuals | 1.44 (1.30-1.60) | <0.001 | 0.50 (0.35-0.73) | <0.001 | 1.26 (1.01-1.58) | 0.043 | 1.59 (1.31-1.94) | <0.001 | 1.84 (1.52-2.24) | <0.001 | 1.71 (1.28-2.28) | <0.001 |
|  | MSM-IDUs | 0.85 (0.55-1.33) | 0.483 | 0.44 (0.20-0.98) | 0.045 | 0.57 (0.26-1.26) | 0.167 | 1.04 (0.38-2.89) | 0.938 | 2.40 (0.66-8.78) | 0.186 | - |  |
|  | Heterosexuals-IDUs | 1.87 (1.55-2.25) | <0.001 | 1.49 (0.96-2.30) | 0.072 | 2.25 (1.64-3.07) | <0.001 | 1.12 (0.72-1.75) | 0.618 | 0.70 (0.38-1.27) | 0.238 | 1.08 (0.39-3.05) | 0.878 |
|  | Haemophilia /Perinatal transmission | 0.95 (0.51-1.78) | 0.883 | 0.22 (0.07-0.70) | 0.010 | 0.84 (0.26-2.70) | 0.773 | 0.99 (0.21-4.76) | 0.991 | 3.48 (0.19-63.57) | 0.401 | 2.44 (0.53-11.35) | 0.254 |
|  | Unknown | 0.91 (0.53-1.56) | 0.724 | 0.59 (0.09-3.98) | 0.589 | 0.37 (0.10-1.33) | 0.128 | 4.13 (0.34-49.74) | 0.264 | 2.22 (0.76-6.43) | 0.142 | 0.84 (0.34-2.10) | 0.713 |
|  | Others | 1.55 (1.26-1.90) | <0.001 | 0.91 (0.46-1.80) | 0.785 | 1.44 (0.94-2.21) | 0.094 | 1.41 (0.92-2.16) | 0.111 | 2.06 (1.36-3.10) | 0.001 | 1.72 (1.07-2.76) | 0.025 |
| **Period of enrollment** | 1985-1991 | Ref. |  | - |  |  |  |  |  |  |  |  |  |
|  | 1992-1997 | 1.35 (1.22-1.48) | <0.001 | - |  |  |  |  |  |  |  |  |  |
|  | 1998-2003 | 1.06 (0.95-1.18) | 0.323 | - |  |  |  |  |  |  |  |  |  |
|  | 2004-2009 | 0.87 (0.77-0.98) | 0.026 | - |  |  |  |  |  |  |  |  |  |
|  | 2010-2013 | 0.90 (0.77-1.04) | 0.158 | - |  |  |  |  |  |  |  |  |  |

**Supplementary Table 1.** Multivariable logistic regression model: association of demographical and clinical features with advanced HIV disease according to observation period.

Abbreviations: **95%CI**: 95% confidence interval; **OR**: odds ratio; **Ref:** reference category.

**Supplementary Table 2**.

Survival rate at year-1 and at year-5 from 1985 to 2009 according to late presentation and advanced HIV disease (weighted for losses to follow-up).

|  | **Late presentation** | | | | **Advanced HIV disease** | | | |
| --- | --- | --- | --- | --- | --- | --- | --- | --- |
|  | 1-year |  | 5-year |  | 1-year |  | 5-year |  |
| **Period** | no | yes | no | yes | no | yes | no | yes |
| 1985-1991 | 99.4% | 65.6% | 91.9% | 24.1% | 99.3% | 55.8% | 88.3% | 10.6% |
| 1992-1997 | 99.5% | 75.6% | 95.1% | 54.9% | 99.6% | 67.8% | 94.4% | 42.4% |
| 1998-2003 | 99.3% | 93.3% | 97.3% | 87.4% | 99.2% | 91.1% | 96.5% | 84.4% |
| 2004-2009 | 99.1% | 95.4% | 97.3% | 92.0% | 98.9% | 93.9% | 96.7% | 90.5% |
